# Supplementary material for: Exploring African Community Attitudes Towards Mental Illness in Australia: A Cross-Sectional Study
Source: Healthcare (Basel). 2025 Dec 1;13(23):3115. doi: 10.3390/healthcare13233115 (PMC12692506; doi:10.3390/healthcare13233115)
Supplement: Supplementary file 1 [file healthcare-13-03115-s001.zip › healthcare-3877872-supplementary.pdf]

Table S1. Demographic information of participants in the study.

**Variables**	**Frequency**	**Percent**
Male	59	53.6
Female	51	46.4
Total	110	100.0
**Age**		
No response	35	31.8
20-30 years	50	45.5
31-40 years	17	15.5
41-50 years	5	4.5
**Religion**		
No response	4	3.6
Christian	90	81.8
Muslim	16	14.5
Total	110	100.0
**Region**		
No response	6	5.5
West Africa	102	92.7
East Africa	1	.9
North Africa	1	.9
Total	110	100.0
**Country of origin**		
No response	63	57.3
Nigeria	44	40.0
Kenya	3	2.7
Total	110	100.0
**Marital status**		
No response	2	1.8
Married	43	39.1
Single	64	58.2
Divorced	1	.9
Total	110	100.0
**Have children**		
No response	5	4.5
Yes	39	35.5
No	66	60.0
Total	110	100.0
**Years in Australia**		
No response	97	88.2
1-20 years	5	4.5
21-30 years	6	5.5
31-40 years	2	1.8
Total	110	100.0

**Highest educational level**		
No response	11	10.0
Never attended school	5	4.5
Primary school	2	1.8
Secondary school	4	3.6
TAFE/College	3	2.7
Tertiary	85	77.3
Total	110	100.0
**English Proficiency**		
No response	4	3.6
Very good	65	59.1
Good	36	32.7
Average	4	3.6
Little	1	0.9
Total	110	100.0
**Current work status**		
No response	9	8.2
Unemployed	46	41.8
Employed	54	49.1
Retired	1	.9
Total	110	100.0
**Employment type**		
No response	32	29.1
Professional	46	41.8
Semi-professional	23	20.9
Unskilled/labour	9	8.2
Total	110	100.0
**Awareness about people with mental illness**		
No response	5	4.5
Yes	37	33.6
No	68	61.8
Total	110	100.0

Table S2. Pearson Product Moment Correlation showing the relationship between authoritarian, benevolence, social restrictiveness and mental health ideology on community attitude towards mental illness.

	Mean	SD	CAMI	Authoritarian	Benevolence	Social restrictiveness	Mental health ideology
Community attitude towards mental illness	**103.10**	**9.41**	**-**	**.519****	**.411****	**.223***	**.461****
Authoritarian	**26.05**	**5.07**		**-**	**-.106**	**.277****	**-.201***
Benevolence	**17.93**	**5.86**			**-**	**-.494****	**.253****
Social restrictiveness	**31.78**	**6.25**				**-**	**-.413****
Mental health ideology	**27.35**	**6.45**					**-**
